# Lumen-apposing metal stent-induced perforation during endoscopic ultrasound-directed transgastric retrograde cholangiopancreaticography managed with an endoscopic suturing device

**DOI:** 10.1055/a-2362-0806

**Published:** 2024-08-07

**Authors:** Enrico Palmeri, Pieter Hindryckx

**Affiliations:** 1 60200 Gastroenterology, University Hospital Ghent, Ghent, Belgium

A 37-year-old woman was referred to our unit because of typical biliary colic with transient cholestasis. Her relevant medical history included a cholecystectomy and a sleeve gastrectomy, which was subsequently converted to a gastric bypass. After multidisciplinary team (MDT) discussion, we opted for an endoscopic ultrasound-directed transgastric endoscopic retrograde cholangiopancreatography (ERCP; EDGE) using a 20/10 lumen-apposing metal stent (LAMS).

Owing to the patient’s previous sleeve gastrectomy, the LAMS had to be placed between the jejunum and the excluded antrum. As per recently published international expert recommendations, the ERCP was scheduled 2 weeks post-LAMS placement to allow for tract maturation 1 . Nevertheless, the proximal flange of the LAMS dislocated during duodenoscope introduction and ended up in the peritoneal cavity ( Fig. 1 **a,b** ; Video 1 ). A decompressing needle was placed and the proximal flange was endoscopically retrieved from the peritoneal cavity under fluoroscopic guidance. After an unsuccessful attempt had been made to reposition the proximal flange in the jejunum ( Fig. 1 **c** ), a guidewire was placed through the LAMS lumen into the excluded antrum. A bridging attempt using a 22-mm × 10-cm fully covered enteral stent through the LAMS lumen also failed ( Fig. 1 **d** ). Subsequently, intraperitoneal endoscopic closure of the perforation in the excluded antrum was performed with the X-tack system (Boston Scientific, Massachusetts, USA) ( Fig. 1 **e** ). Next, we removed the LAMS and the enteral stent from the peritoneal cavity and completely sealed the fistula tract from the jejunal side with X-tack endosutures and clips ( Fig. 1 **f,g** ). Complete closure of the jejunal perforation was confirmed with contrast enterography ( Fig. 1 **h** ).

**Fig. 1:**
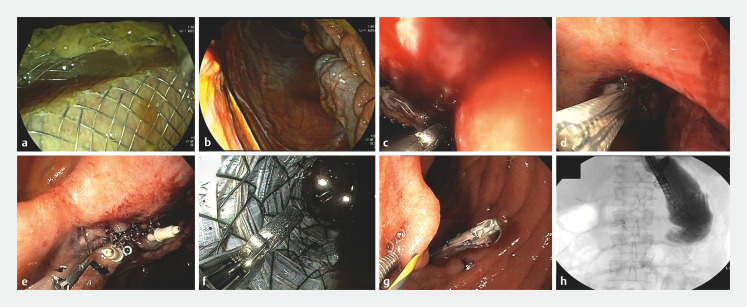
Endoscopic images showing: **a** the duodenoscope being introduced through the lumen-apposing metal stent (LAMS); **b** migration of the LAMS into the peritoneal cavity; **c** an unsuccessful attempt to reposition the proximal flange of the LAMS into the jejunum; **d** an unsuccessful attempt to bridge the excluded stomach and the alimentary jejunal limb with a covered enteral stent; **e** intraperitoneal endoscopic closure of the perforation in the excluded antrum using an endoscopic suturing system and one clip; **f** removal of the enteral stent and the LAMS; **g** closure of the jejunal perforation with the endoscopic suturing system and clips; **h** fluoroscopic image during contrast enterography confirming complete sealing of the jejunal perforation.

Iatrogenic perforation resulting from a lumen-apposing metal stent that had migrated intraperitoneally during endoscopic ultrasound-directed transgastric endoscopic retrograde cholangiopancreatography is managed with a novel dedicated endoscopic suturing device.Video 1

The patient was put on antibiotics and was able to start oral feeding the day after the procedure. Other than moderate abdominal pain for the first 2 days after the procedure and a transient elevation of the inflammatory markers, her further clinical course was uneventful. After repeat MDT discussion, she was scheduled for a laparoscopy-assisted ERCP, during which her biliary disease was treated successfully.

This case illustrates the usefulness of endoscopic suturing devices as minimally invasive alternatives to surgery for management of large iatrogenic perforations.

Endoscopy_UCTN_Code_CPL_1AL_2AD

## Notes

Endoscopy E-Videos https://eref.thieme.de/e-videos
*E-Videos* is an open access online section of the journal *Endoscopy* , reporting on interesting cases and new techniques in gastroenterological endoscopy. All papers include a high-quality video and are published with a Creative Commons CC-BY license. Endoscopy E-Videos qualify for HINARI discounts and waivers and eligibility is automatically checked during the submission process. We grant 100% waivers to articles whose corresponding authors are based in Group A countries and 50% waivers to those who are based in Group B countries as classified by Research4Life (see: https://www.research4life.org/access/eligibility/ ). This section has its own submission website at https://mc.manuscriptcentral.com/e-videos .
